# Overview of international teledermatology

**Published:** 2010-07-20

**Authors:** Brijal Desai, Karen McKoy, Carrie Kovarik

**Affiliations:** 1 University of Pennsylvania School of Medicine, Philadelphia, Pennsylvania, US, 2 Lahey Clinic, Boston, Massachusetts, US; 2 Lahey Clinic, Boston, Massachusetts, US

**Keywords:** Telemedicine,, teledermatology,, teleconsultation,, Internet

## Abstract

Teledermatology is essentially “dermatology at a distance”, using one of many communication technologies to expand the reach of a dermatologist to those in need of their specialized knowledge. Most international teledermatology is store-and-forward in nature, a method in which images are stored on a computer and then transmitted electronically to a consulting dermatologist. This system is more convenient and less costly than real-time teledermatology. This review will focus on several of the store-and-forward teledermatology systems being developed and utilized successfully internationally. This discussion of “who” is practicing teledermatology is not comprehensive, but attempts to show some of the breadth of teledermatology practice around the world, including government national health plans, commercial endeavors, and charitable work by individuals and institutions. The goal in many instances is to provide better health outcomes through increased access, efficiency, and/or cost-effectiveness. More studies ultimately need to be conducted to develop a more comprehensive and sustainable model for teledermatology.

## Introduction

Telemedicine (medicine practiced at a distance using communication technology) has been a slowly growing field. Dermatology is particularly suited for telemedicine, as it is visual in nature. Interest in teledermatology (TD) is growing due to changing consumer demands, inequity in access to specialty care, the limited supply of dermatologists, and technological advances. Numerous reviews regarding the feasibility, accuracy, and acceptability of TD have been published [1-3]. TD has been adopted more for use in the developed world, most often in specific situations where the medical care delivery systems find it fills a gap in specialty care and is cost-effective. However, TD offers even more potential benefits to those in the developing world that lack even basic health care access.

### Definition and Overview of Teledermatology

TD is essentially “dermatology a distance,” using one of many communication technologies to expand the reach of a dermatologist to those in need of their specialized knowledge. Most interactions to date are doctor-to-doctor, but some programs are offering interactions directly with patients. It should be stressed that the same technologies may be multipurpose, serving also as an education tool. The primary requirements include a simple interface, sufficient communication systems, and trained users. TD today is performed by one of two main methods. The first uses “real-time” (synchronous) videoconferencing, with the patient and dermatologist meeting simultaneously but in different locations, usually with the referring healthcare provider present with the patient. The second is a “store-and-forward” (asynchronous) method, in which images are transmitted electronically to a consulting dermatologist, such as via email or a web-based interface. In addition, there are hybrid models integrating aspects of videoconferencing with store-and-forward (SF) systems which are designed to increase diagnostic reliability. However, a recent prospective clinical trial by Romero, et al., has shown that when training in digital photography is standardized, the diagnostic reliability of a hybrid system with audio is no better than SF alone [4]. Most international TD is store-and-forward in nature and this review will focus on this modality, which is more convenient and less costly than real-time TD. There are now many guidelines for practicing TD available; the most practical and technical standards have been promulgated by the American Telemedicine Association TD Special Interest Group [5]

**Figure 1:**
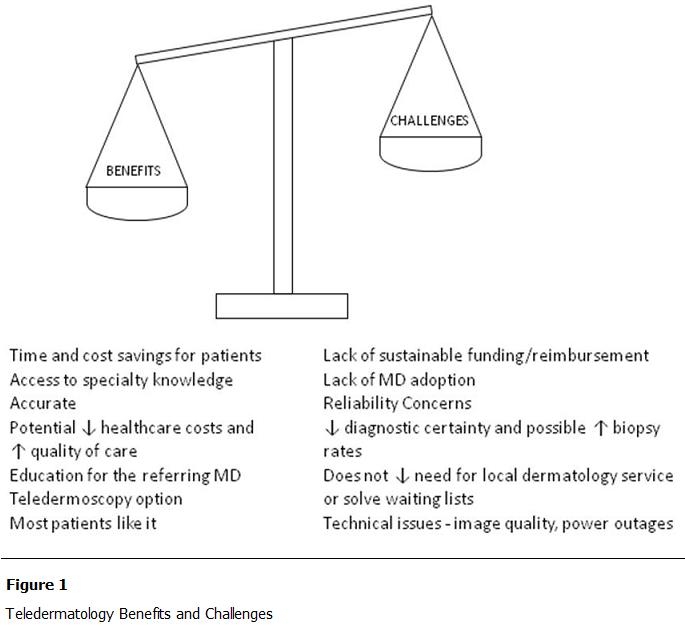
Teledermatology Benefits and Challenges

Figure 1 details the benefits and challenges of using TD. Benefits include the fact that patients generally approve of its use, although there are always some who prefer face-to-face interaction. For those in remote or under-served areas, it offers travel time and cost savings, as well as access to otherwise unavailable expertise. Given an adequate history and sufficient image data, TD consultations result in highly reliable diagnoses that compare favorably with conventional clinic-based care [6-9]. Although a face-to-face visit with the specialist is preferred and is the “gold-standard”, many patients never have the chance to see a dermatologist, and non-dermatologists have a lower diagnostic accuracy level for skin disease [10]. In addition, because the opinion is generally delivered in a timely manner, there is high educational value for the referring physician.

There are several challenges affecting the adoption of TD, including lack of funding for the infrastructure, lack of reimbursement for the referring or consulting physician, slow adoption of new techniques by physicians (who are a generally conservative group), concerns regarding malpractice and licensure, the possibility that biopsy recommendations may be higher because of an increase in diagnostic uncertainty, and technical issues such as image quality. The need for face-to-face care will always remain for some patients. In terms of TD adoption in developing nations, there are further issues of varying regional healthcare practices, priorities, and policies.

## Evaluations of TD

A growing body of knowledge has demonstrated the utility of TD in decreasing time to receiving specialty care and the utility of TD in triaging patients. However, more outcome and cost-effectiveness studies need to be conducted to support the broader adoption and implementation of TD. In terms of clinical outcomes, several store-and-forward studies have shown that patients can be “seen” with less delay. In one study, the time to be seen for the TD group was a median of 41 days, compared to 127 days for the face-to-face group [11]. In another study, dermatologists  responded to store-and-forward consultations in an average of 2.17 days, compared with a mean wait time of 90 days for clinic-based appointments [12]. Two studies confirm that the clinical courses of TD patients and traditionally seen patients are similar [13,14]. Multiple studies have evaluated the proportion of store-and-forward TD consults that avoided, or would have avoided, a clinic visit to a dermatologist (ranging from 18-58%) and cutting in half the number of “urgent” referrals (from 64% to 32%) [15-21]. A study by Whited et al found that dermatologists required an average of 7.2 minutes to review a store-and-forward TD consult compared to 24.4 minutes for clinic-based visits [22]. The challenges of assessing outcomes in TD include patient loss to follow-up with the teledermatologist, lack of information in patient records, and low rates of patient return to the referring clinician for follow-up.

Economic studies of TD are scarce. It has proven difficult to demonstrate that TD results in economic savings, as teleconsultation does not fit into current models of care. More evaluation studies with a focus on clinical outcomes, such as preventable referrals or time to recovery, are needed to prove that TD is a cost-saving technology.

## Rationale for TD in under-served areas

**Figure 2:**
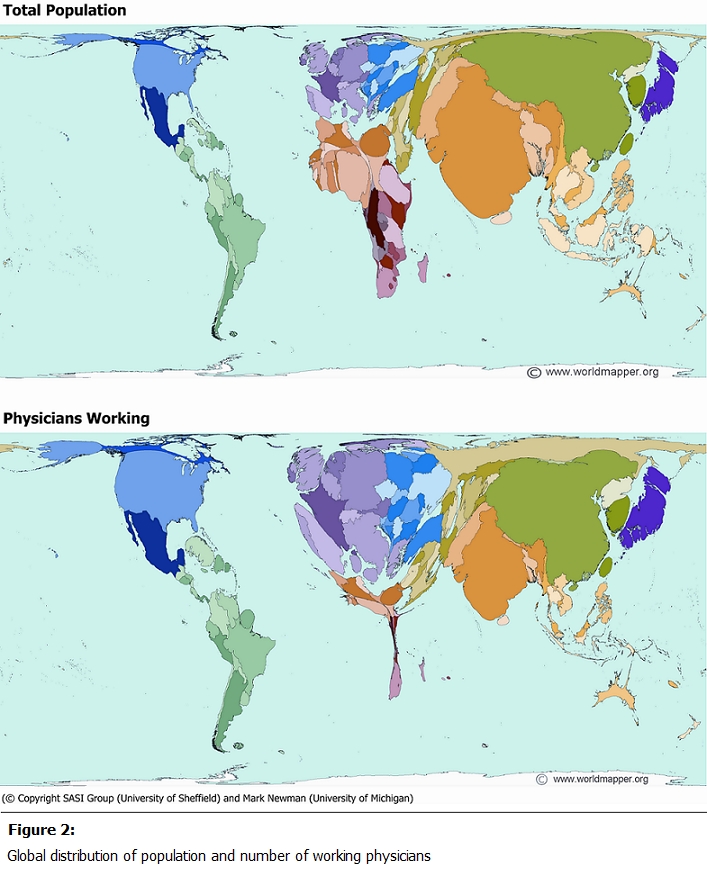
Global distribution of population and number of working physicians

A recent popular book by TL Friedman, “The World is Flat” [23], assumes that globalization, in large part made possible by new communication technology, will make the world more prosperous and equal, as well as transparent and democratic. Unfortunately, these technologies have not changed the way economic, social, and political power is exercised nationally and internationally. Figure 2 shows maps representing the world’s population and physician distribution. In 2004, there were 7.7 million physicians working around the world; if physicians were distributed according to population, there would be 124 physicians to every 100,000 people. However, half of physicians live in territories with less than a fifth of the world’s population. The fifth of the world’s population that is the most economically challenged is served by only 2% of the world’s physicians [24]. This physician maldistribution is further pronounced in specialty care such as dermatology. The International Foundation for Dermatology estimates that 3 billion people living in more than 100 countries lack basic care for skin diseases [25]. In addition, there may be a greater need for this care, as surveys have shown that up to 80% of people in developing countries suffer from skin diseases [26], in contrast to 52% of people in a UK study [27].

The ideal dermatologist to population ratio has been estimated to be 1:50,000 [28]. A significant problem in most countries is the rural-urban distributional imbalance of healthcare workers, especially specialists, such as dermatologists. For example, there are nearly 5000 dermatologists in the Indian Association of Dermatologists, Venereologists and Leprologists [29], theoretically equaling a ratio of 1 dermatologist per 226,000 people; however, in India, 72% of 1.2 billion people live in villages and virtually all the dermatologists are in cities. This actually equates to 1:50,000 in the cities and none for 867 million rurally. In Peru, 200 of the country’s 250 dermatologists practice in Lima, where a third of the population resides [30]. Table 1 gives estimates of this rural to urban maldistribution in the developing world. Even in urban areas, there is another divide between the urban rich and poor in access to skincare.

Recognizing the gross misalignment of health care resources to actual need, particularly in dermatology, it is the hope of many that technology could at least partially bridge the gap and make the world a bit flatter, or more equitable. The benefits that teledermatology can bring to the developing world are the same as discussed previously for TD in general.

## Examples of International TD

What is international TD? At the first World Congress of the International Society of Teledermatology [31], offerings from the developed world covered American Indian Health Service dermatology care in Alaska, military medicine in Serbia, teledermoscopy, remote triaging for skin cancer, and home care and follow-up of previously seen dermatology patients. Most of these interactions were physician to physician consultations and stayed within one country; however, some were direct physician to patient interactions. Presentations at the World Congress of TD from developing countries were physician to physician interactions and, in most cases, were between physicians in different countries. Usually the programs in developing countries were for educational or charitable purposes, rather than as an integrated part of a medical care delivery system.

Most users of TD are institution based, as development of systems for communication, coverage, and record keeping is easier. However, it must be recognized that a number of individual practitioners practice TD informally and pro bono or on physician case-sharing and education websites, usually on a doctor-to-doctor basis. This discussion of “who” is practicing TD is not comprehensive, but attempts to show some of the breadth of TD practice around the world. Most of these systems have been developed with the aim to provide greater access to dermatological care in resource limited or underserved areas, however, as outcome studies demonstrate the increased utility of TD, the applications of TD will continue to evolve over time. Much of this information is readily available on the Telemedicine Information Exchange website [32].

### Government National Health Systems (including military)

Governments who currently oversee nationalized healthcare systems are in constant need of increasing access to specialty care and decreasing overall healthcare costs. Telemedicine is one such attempt to help accomplish these tasks. In the setting of national healthcare systems, TD has emerged as a potential solution to help with the triage of patients and expand specialty care.

Examples of national health systems delivery of TD can be found in the United Kingdom, Canada, Norway and Saudi Arabia. Over the last 10 years in the UK, the National Health Service (NHS) has sponsored TD services that are mostly based on store-and-forward systems, with some operating as nurse or general practitioner clinics with a link for consultant review and/or triage. In a recent study that interviewed various stakeholders in TD development within the NHS -UK, the authors concluded that the original policy vision of TD as an answer for long waiting lists and consultant shortages was not realized. They also found that TD services were initially viewed as a diagnostic service but then increasingly gained value as a triage and management service. For example, in a recent prospective study at a skin cancer clinic, it was shown that a larger proportion of patients with melanoma and squamous cell carcinoma (SCC) were seen earlier by dermatologists when using a TD system [33]. The TD services which continued were those for which the perceived benefits, for example, saving patients’ and/or health professionals’ traveling time and costs, or reducing waiting times, clearly outweighed the effort and commitment required to make the system work in the local setting. The issue of the need for widespread and ongoing support by all of those contributing to such service reconfigurations was a recurring theme [34]. Information regarding the use of TD in the UK is available from the website of the British Teledermatology Society [35].

In Canada over 25% of the population is in remote and difficult to reach regions; therefore, telehealth development has focused on the health care needs of rural, remote, and northern communities. In addition, they have focused on improving access to the universal health care system for First Nations and Inuit communities [36]. Alberta [37],British Columbia [38], Nova Scotia [39] and Ontario [40] have large and well-developed provincial telehealth networks. Nova Scotia and Ontario, specifically, offer teledermatology services using live videoconferencing from a telemedicine studio. Patient wait times for a non-urgent dermatology visit in Nova Scotia were reported for 2009 as 13.7 weeks for a face-to-face consult with a dermatologist versus 4.6 weeks for a teledermatology consult [41].

In 1996, Norway was the first national health system to reimburse for telemedicine. The Norwegian Centre for Telemedicine is government funded, with the primary aim to serve the public Norwegian Health Service. In 2001, 19,000 patients in Northern Norway alone were diagnosed and treated by doctors through the use of telemedicine; TD is one of the priority areas for expansion [42].

Telemedicine and teledermatology have been widely practiced in the US military, particularly the army, for several years [43]. A US Army TD program has performed over 30,000 consultations nationally and internationally since inception [44]. In addition, the Armed Forces Institute of Pathology runs a telepathology service. Experience in the US Veteran’s Administration is that TD can result in treatment initiation significantly sooner than patients receiving usual care and avoid the need for a face-to-face dermatology clinic appointment in 18.5% of patients. This system also provided evidence that TD is cost-effective and decreased the time required for patients to reach a point of initial definitive care [45]. Other  countries whose military use TD include Finland [46], Israel [47], India [48] and China [49].

### Other Government Initiatives

Several government sponsored telehealth services have been created to address underserved remote regions and populations. TD is often included among these services. Most of these programs are focused on expanding services to patients residing in their respective countries.

The Alaska Federal Health Care Access Network (AFHCAN) is a very busy and successful federal telehealth initiative operating in Alaska; the number of users and clinical services has grown significantly over the years and now connects more than 700 providers caring for more than 300,000 Federal beneficiaries spread over 250 sites. More than 90% of these patients do not have access to the Alaskan road system. Under the guidance of Dr. John Bocachica, TD is an active component of this program.

With the aid of HealthSAT, India’s telemedicine initiative of the Indian Space Research Organization (ISRO), more than 25,000 patients have so far been provided with teleconsultation and treatment. Connectivity is given free of charge to government entities. An impact study conducted on a thousand patients has revealed that there is a significant cost saving in the system since the patients avoid expenses for travel, stay, and for treatment at the hospitals in the cities. ISRO has established the facility in nearly 60 remote hospitals, which have been connected with 20 specialty city hospitals.

The Australian New South Wales Telehealth Initiative serves a variety of sites, including aboriginal settlements, prisons, nursing homes, and trauma centers. In 2004, the Australian College of Rural and Remote Medicine in a joint initiative with Queensland Divisions of General Practice set up TD with funding from the Commonwealth Department of Health and Ageing under the Medical Specialist Outreach Assistance Program. This service provides teleconsultation and online education in dermatology to doctors Australia wide [50].

Clinicians at the University of Washington in the US, including Dr. Roy Colven, in collaboration with the University of Cape Town in South Africa, worked on a South African government Medical Research Council TD pilot. Within the public sector of health care which covers the majority of the population in South Africa, there is an average of one dermatologist for 3-4 million people. The pilot study demonstrated a reduction in the burden of skin disease and improved quality of health-care practice in remote areas, but data demonstrating the extent of sustainable benefits derived from teledermatology support are currently insufficient.

### Commercial

There are a few commercial teledermatology services. There is no available information on the reliability, cost-effectiveness, or quality of almost all of these endeavors and mention of them in this article is not to be considered an endorsement. In addition, it is unknown as to whether commercial initiatives will result in a substantial increase in patient access to dermatological services. One of the first commercial offerings was TDS Telemedicine, which established its TD business in the United Kingdom and operated under contract for the NHS beginning in 1996; it announced plans to expand to the US in 2003, but was not successful and is no longer operating.

One of the largest commercial teledermatology ventures has been the KSYOS Telemedical Centre in the Netherlands. The Netherlands health care system requires all to enroll in insurance plans, which reimburse the referring physicians, the dermatologists, and KYSOS for teledermatology consults. The business provides software, hardware, education, support, quality monitoring, administration and billing services. They state that over 2000 referring general practitioners have referred over 25,000 patients to 164 dermatologists in their regions through teledermatology. They also claim a 65-70% reduction in referrals to dermatologists for in-person visits, as well as significant cost-reductions for the health system [51].

TeleDerm Solutions is a US based company, which aims to provide cost-efficient online dermatology consultations for healthcare organizations and private physicians around the world. Customers include the University of Miami School of Medicine and the Veteran’s Hospital in Georgia. In their business model, they suggest that the primary care referring doc, as well as the consulting dermatologist, be paid a fee and that a consult manager is needed on both sides.[52] Finally, there are some enterprising individuals offering online consultations in dermatology directly to consumers for a fee. One example is LivePerson, which provides an online service facilitating real-time or email assistance and expert advice; seven consultants were recently listed for dermatology [53]. Another is iDoc24 based in Sweden, offering anonymous dermatology advice for a fee using a cellular phone [54].

### Non-Profit and Charitable Services

Many non-profit institutions and individuals have been involved in telemedicine projects, including, but not focusing solely on TD per se. These initiatives have been developed to provide high level specialty care to developing countries and resource limited settings, typically outside of their country of origin. In certain instances, these efforts have led to the development of online communities which facilitate the sharing of complex cases for consultation and educational purposes.

The Institute of Tropical Medicine, in Antwerp, Belgium aims to introduce high-quality care for HIV/AIDS patients living in resource limited settings [55]. With over 7000 website visits a year, consultations are frequent from South Africa and Cambodia. This platform aims to provide training, medical decision making support, and education to health care providers working in these settings.

The Swinfen Charitable Trust was set up with the aim of assisting people in the developing world by establishing telemedicine links between hospitals in the developing world and specialists who generously give free advice by e-mail. The Trust has expanded since starting in 1999 to 136 remote hospitals with access to nearly 410 consultants covering around 130 medical and surgical specialties. In the first 7 years of operation, over 1500 referrals were submitted in a wide range of specialty areas. The median length of time required to provide a specialist’s response was 1.8 days. Five hospitals submitted cases for more than four years; however, their activity data showed a trend in declining referral rates over the four-year period, which may represent successful knowledge transfer.

The American Academy of Dermatology (AAD) has been interested in international volunteer efforts. Most of the opportunities presented for members have been through the Education and Volunteers Abroad Committee, primarily as direct educational programs through Health Volunteers Overseas. Recently, the Teledermatology Outreach Committee, within the Telemedicine Task Force, has developed a teledermatology project in Africa (Africa.telederm.org) in collaboration with several partners, including the Medical University of Graz, the Baylor International Pediatric AIDS Initiative (BIPAI), the Botswana UPenn Partnership (BUP), and many other private and public organizations that provide health care throughout Africa. The Africa Teledermatology project has been created to provide dermatology support to local physicians, dermatologists, and health care workers in hospitals and clinics throughout Africa. This support is provided through TD consultation services, discussion pertaining to diagnosis and management of patients with skin diseases, links to educational resources, and access to a dermatologic curriculum created specifically for African sites. To date, over 700 consultations have been submitted from over twelve African countries, with the most consultations coming from Botswana, South Africa, Swaziland, Lesotho, Malawi, Uganda, Tanzania, and Liberia. Representative diagnoses include general dermatologic diseases, such as tinea corporis, impetigo, drug reactions, atopic dermatitis, as well as HIV-related conditions including Kaposi’s sarcoma, human papillomavirus (HPV) related cutaneous malignancies, disseminated fungal infections, and pruritic papular eruptions.

Given the success of the Africa Teledermatology Project, several other programs have been developed using the same web-based platform. Proyecto Latinoamericano de Teledermatología (latinoamerica.telederm.org) is a similar site, translated into Spanish, which serves the Latin American community. In addition, LEPROSY & Global Dermatology (leprosy.telederm.org), a collaboration project between Graz (Austria), Genoa (Italy), Brisbane (Australia) &  Manaus (Amazonas – Brazil), is a unique medical web application whereby dermatologists, pathologists, dermatopathologists, infectious disease specialists, general practitioners, residents, students in medicine, and any other healthcare workers interested in tropical dermatology can discuss interesting and unusual cases in clinical dermatology and dermatopathology of tropical diseases with special emphasis on leprosy. The website can be viewed in English, Spanish, and Portuguese, allowing for expanded collaborations. All of the telederm.org projects are free of charge, and only require the submitting clinician to register on the website for use.

Other institutions with teleconsultation services (usually to developing countries) include the Center for Connected Health (a division of Partners HealthCare in Boston), University of Nebraska Medical Center, the University of Transkei in South Africa, the Centro de Telemedicina in Colombia, and the telepathology network of the University of Basel (iPath).

Individual physicians have been sharing cases and receiving informal TD consultations from colleagues for many years. One of the larger groups is The Community for Teledermatology (www.telederm.org), a free online community of physicians allowing doctor-to-doctor consultations. This group was established in 2002 at the University of Graz in Austria [56]. There are now over 600 physicians registered from over 50 countries; over 3000 teleconsultations have been posted. Similar communities of dermatologists also exist in Iran [57], Pakistan [58], and Russia [59]. Another physician case-sharing site run by Drs. David Elpern and Henry Foong posts approximately one complicated case per month and has about 400 physician participants [60].

The Australasian College of Dermatologists has a Yahoo Group called DERMO, which has 175 faculty members participating out of 300 total Australian dermatologists. This has been very successful in providing an area for discussion of difficult clinical cases. Averaging 200 monthly, submitted cases and questions and answers automatically go out by email to all registered members of the group. The Skin Cancer Society in Australia also has a dermoscopy blog used by its members to share opinions on dermoscopy cases; there are more than 1100 cases with over 3500 images.

## Challenges for International TD

The goals of reaching the underserved are lofty, yet teledermatology is by no means the perfect answer. By utilizing TD, we may be able to reach some of the underserved, yet the fact remains that the infrastructure and manpower to deliver good dermatology care is inadequate even in many developed countries. Obstacles limiting more widespread introduction of telemedicine services in the developed world include human acceptance, technology factors, the lack of health outcomes data, and legal and reimbursement issues. In addition, any service needs to be tailored to meet patient and physician needs and expectations in a particular location. A solid evidence base and a thoughtful implementation strategy are required to ensure doctors are motivated to participate in telemedicine initiatives.

Specific challenges related to TD in the developing world include timeliness of the consultation, the lack of feedback to the consultant, resistance to “foreign” methods or suspicion regarding data collection by foreigners, the fact that consultant recommendations may not be available or affordable, and region-specific regulatory and health policy issues.

Another challenge to using technology in developing countries is the still very uneven penetration of internet access to a large part of the world. Uptake of information and communication technology (ICT) globally continues at an uneven pace, and the “digital divide” remains a significant obstacle to achieving global development goals.The digital divide is understood broadly to be the gap between those with access to ICT and its benefits and those without it. This issue is specifically acknowledged in the United Nations Millennium Development Goals. Table 2 shows current global internet penetration data.

**Table 1:** Dermatologists—World Distribution

Country	Urban (Dermatologist/People)	Rural (Dermatologist/People)
India	1:50,000	0:847,000,000
Asia (11 countries)	1:200,000	1:78,000,000
Sub-Saharan Africa (7 countries)	1:1,000,000	0-1:5 to 50,000,000
Central/S. America (10 countries)	1:76,000	0-1:1 to 66,000,000

Source: Dyall-Smith, D., Marks, R. Dermatology at the Millennium: The Proceedings of the 19th World Congress of Dermatology - Informa Healthcare - 1850700052 - 1999

**Table 2:** Internet Penetration as of March, 2009

Country	% of Internet Users/Population
World	23.8%
N. America	74.4%
Europe	48.9%
S. America	29.9%
Middle East	23.3%
Asia	17.4%
Africa	5.6%

www.internetworldstats.com

## Future Directions

Although the many teledermatology programs detailed here reach a large community of users throughout the world, use of the service still requires access to a computer with a sufficient internet connection. Lack of computers with internet connectivity is one of the most significant barriers that prevents scale up of store-and-forward teledermatology to the most underserved areas of the world. However, many of these regions are supported by significant cellular phone coverage, with the majority of the world’s cell phone subscriptions now arising in the developing world.  As such, mobile teledermatology, or the use of specialized mobile phones to perform store-and-forward teledermatology, may quickly become  instrumental in expanding the role of dermatology in the global health arena. One non-profit group that is developing mobile teledermatology in the developing world is ClickHealth [61]. The africa.telederm.org group, under program director Dr. Carrie Kovarik, has successfully piloted mobile teledermatology in Botswana, Ghana, and Egypt, with plans for continued expansion within Africa.

In addition to the development of infrastructure and increase of manpower, there are also significant regulatory and health policy hurdles to overcome to fully realize the potential of telemedicine and TD. Health policy largely remains the sovereign domain of individual countries. For example, Malaysia and several other countries only allow those holding a license to practice in their country to provide care to their citizens. Specific country “e-health” plans and policy have focused primarily on information and communication technology, rather than health care needs. In addition, many developing countries suffer from a multiplicity of donors with differing agendas, dependence on institution-to-institution agreements, and on volunteer efforts [62]. However, to be effective, global e-health must become fully integrated into existing national and international health-related structures. This will only be achieved through implementing globally accepted strategies, principles, and complementary policy options that are supported by health outcome and cost-effectiveness studies. The Rockefeller Foundation sponsored the "Making the eHealth Connection" conference with international partners from leading health organizations in 2008. The recommendations of this conference resulted in the “Bellagio eHealth Call to Action”, representing stakeholders’ commitment to improve health and decrease disparities in health care worldwide through sharing of appropriate practices, building capacity, and promoting innovative eHealth solutions. This document will be submitted for review and consideration to global institutions, individuals, and governments with the power to change policy and practice [63].

## Competing interests

The authors have no conflicts of interest to report.
